# Numerical simulation and experimental research on optimization of pneumatic convey system in pneumatic seeder

**DOI:** 10.1371/journal.pone.0338016

**Published:** 2025-12-22

**Authors:** Fang Guo, Kaiqi Liu, Genhao Liu, Senhao Wang, Zhanfeng Hou, Jiacheng Li, Yu Ma

**Affiliations:** 1 School of Mechanical Science and Technology, Huazhong University of Science and Technology, Wuhan, China; 2 School of Mechanical and Electrical Engineering, Inner Mongolia Agricultural University, Hohhot, China; 3 Engineering Research Center for Full-Process Intelligent Equipment in Forage Feed Production of Inner Mongolia Autonomous Region, Hohhot, China; 4 School of Mechanical and Electrical Engineering and Automation, Shanghai, China; Koneru Lakshmaiah Education Foundation / Indian and Xidian University, INDIA

## Abstract

The use of pneumatic seeders for coated grass seed effectively addresses the challenge of seed establishment in arid and semi-arid regions, significantly contributing to the mitigation of soil desertification. However, during pneumatic seeder operations, high airflow velocities can lead to increased seed breakage rates, while low airflow velocities may result in blockages. To address these issues, this study employs both experimental and simulation methods to optimize the pneumatic conveying system of the seeder. A theoretical model of the pneumatic conveying system was developed to analyze the velocity field, pressure field, and seed trajectory within the flow field. The study focused on optimizing three parameters: Diameter of the throat outlet, diameter of the throat inlet, and the seed inlet angle. The key findings are as follows: (1) An optimized throat diameter of *D*2 = 58 mm is less prone to blockage; (2) Optimization of the neck ratio does not effectively mitigate the blockage issue; and (3) A seed inlet angle of *α* = 77° reduces seed breakage. This research elucidates the mechanisms of airflow and seed distribution within the pneumatic conveying system, providing solutions to minimize seed breakage and blockage, thereby enhancing the design and broader application of pneumatic seeders.

## Introduction

Grasslands are the largest terrestrial ecosystem type in the world, covering 40% of the land area [1,2]. Grassland is an important ecological barrier, a green barrier in arid and agro-pastoral areas, and a source of feed for the modernization and development of the regional livestock breeding industry. In terms of ecological function, it can maintain soil and water, prevent wind and sand, etc. [3–5]. In recent years, due to extensive reclamation and overgrazing have severely damaged grasslands, leading to resource degradation, increasing desertification, salinization, rocky desertification, and decreasing the ecological carrying capacity of the grasslands [6–8]. Grassland degradation and desertification have damaged their ecological defense and economic output functions. Therefore, it is crucial to explore methods to enhance the grassland ecological environment and promote sustainable development, creating a positive feedback loop for both ecological and economic benefits [9–11].

**Fig 1:**
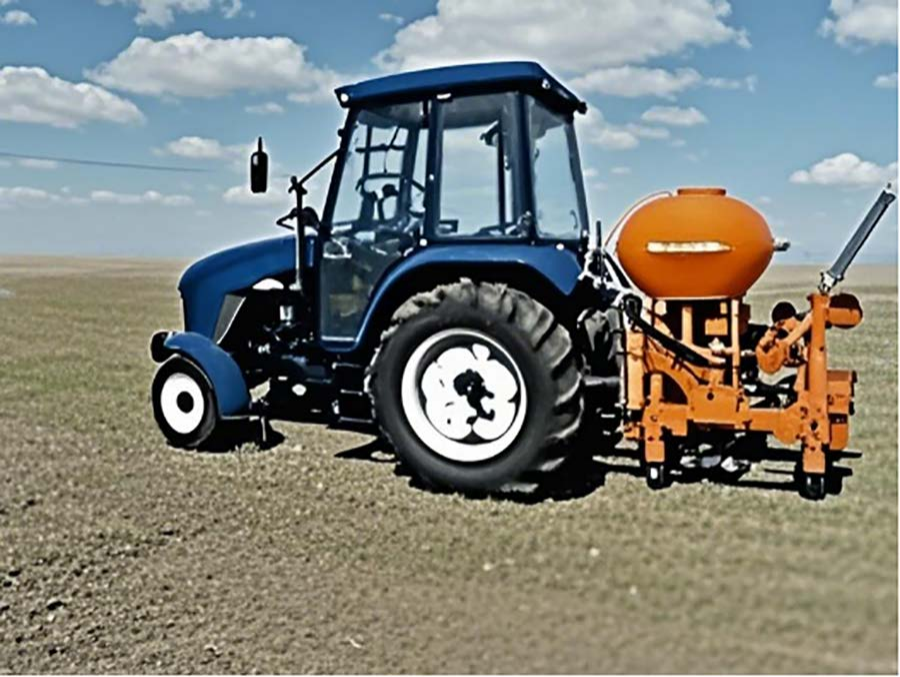
Pneumatic seeders at work.

In 1960, Japan imported hydraulic pneumatic seeding technology from the United States and applied it to road embankment slopes. Since then, this seeding method has played a crucial role in ecological protection and vegetation restoration [12]. In the 1980s, pneumatic row-planting seeder became widely used in the United States, Europe, and Australia [13]. In the 1990s, the French company Monosem developed the NGPLUS pneumatic precision planter, featuring a seed dispenser using multi-material coupling technology [14]. And then later by L.G.L. Copp et al studied A precision seeder operated by suction [15]. And E.M. Zubrilina and O.S. Babenko with the development of an automated control and management system for seeding in rowed seed drills [16]. The operational schematic of the pneumatic seeder is illustrated in Fig 1.

The previously studied seeder, while effective for general seeding purposes, is found to be unsuitable for grassland restoration due to several limitations. Primarily, its design is tailored for crops rather than the diverse vegetation characteristic of grasslands, and its precision operation does not align with the distribution requirements of grassland plants. Furthermore, the machine’s operational mode can inadvertently disrupt the surface soil, thereby compromising the natural soil environment. Consequently, although it is suitable for crop planting, it lacks the necessary attributes for large-scale grassland restoration projects.

**Fig 2:**
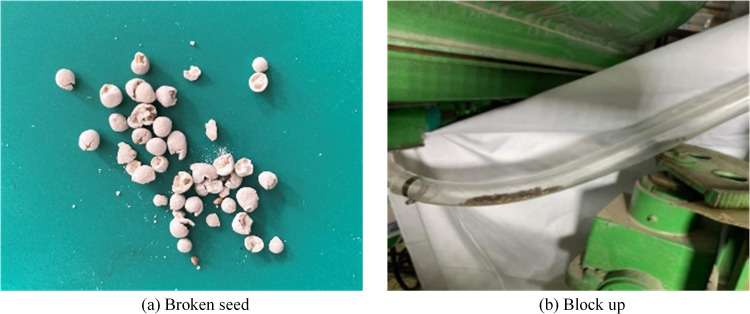
Anomalies in the seed spraying process.

Since its inception in the late 1980s in Shenzhen, China, pneumatic seeder technology has evolved significantly [17]. In the mid-1990s, the development of pneumatic seeder machines by Inner Mongolia Forestry University addressed the need for a large-scale pneumatic seeder in pastures [18]. But there is still a lack of analysis on the pneumatic convey system and the discussion of existing problems, thus Zhang Yun on the main performance of the 4BQD-40 pneumatic seeder design [19]. And the selection of oscillation frequency for the 4BQD-40 pneumatic seeder nozzles was determined through theoretical calculations to minimize seed leakage and optimize replanting area coverage [20]. Mu Houchun designed the 4BQD-40 pneumatic seeder inserted tube jet feeder [21]. Wang Anting’s research on the automatic control system of 4BQD-40 pneumatic seeding machine rows of seeds [22]. Fan Chunmao’s optimization and experimental verification of the oscillation frequency of the pneumatic seeder barrel of the 4BQD-40 vehicle-mounted pneumatic seeder [23]. Researched on automatic variable pneumatic seeder technology of pneumatic seeder and optimization of related parameters based on multi-source information by Chen Yan [24]. Liang Jianping designed a wind-seed mixing negative pressure chamber for a pneumatic seeder intended for shrub seed and sand-fixing agent application, and conducted uniformity tests on its seeding performance [25]. The research done in the above results is mainly for the application of common seeds, but in recent years this pneumatic seeder is more widely used in the grassland vegetation restoration of coated seeds, in the actual application of the seed coating will still have the phenomenon of fragmentation as shown in the Fig 2a below, and in the pneumatic convey system there will be a phenomenon of clogging as shown in the below Fig 2b. Therefore, it is necessary to study the influencing factors of the pressure of each part of the pneumatic conveying system and the influencing factors of the number of collisions of seeds in the conveying process.

This paper investigates the core structure of the pneumatic convey system and optimizes the parameters of the nozzle and seed drop. The flow field is visualized to determine the velocity and pressure distribution, as well as the motion trajectory of seed particles. This research aims to optimize the structure and operational parameters of the pneumatic convey system, providing a theoretical basis for enhancing its efficiency and addressing related issues, thereby facilitating grassland vegetation restoration. This study focuses on optimising the throat structure to mitigate vortex formation and adjusting the seed inlet tilt angle to minimise seed collisions, thereby enhancing the overall efficiency of the seeding process.

## Structure and working principle of pneumatic seeder

This paper explores the optimization of a pneumatic seeding machine’s performance. The machine consists of a frame, high-pressure blower, crank linkage mechanism, amplification mechanism, pneumatic seeder canister swing mechanism, pneumatic seeder canisters, throats, seed boxes, seed discharge mechanism, seed drop, motors, and other components. The device operates as a suction-based pneumatic conveying system, where air and seeds are simultaneously drawn into a throat. The high-pressure blower directs a high-speed airflow into the pneumatic seeder canister drum. At the blower’s outlet, a throat mechanism is strategically positioned to connect to the seed discharge mechanism. This setup enables the seeds to be introduced into the airflow via the throat mechanism, where they become suspended, accelerating through the main airway before being squirted out. Additionally, the pneumatic seeder canister is designed to rotate around a vertical axis up to a maximum angle of 180°, significantly enhancing the machine’s efficiency. This rotational capability allows for more precise and effective seeding, improving overall performance.

**Fig 3:**
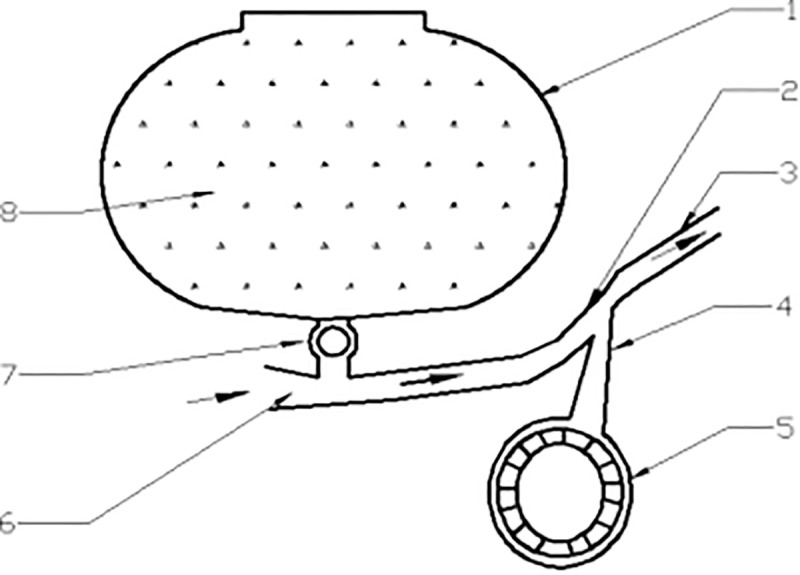
Working principle of pneumatic seeding machine [24]. 1. Seed box 2. Seed delivery tube 3. Pneumatic seeder canister 4. Throat 5. Blower 6. Air inlet hole 7. Seed supply unit 8. Seeds.

As illustrated in Fig 3, the two-dimensional pneumatic conveying system consists of a seed dropping device, throat, and nozzle. The upper part of the seed dropping device is connected to the seed discharging mechanism (7), while the pneumatic seeder cylinder (3) is open to the atmosphere. High-speed air is pumped into the entire pneumatic conveying system at four points by the high-pressure blower, creating negative pressure. This negative pressure allows the seeds entering the seed dropping device to be efficiently drawn into the main air duct.

## Experimental setup

### Experimental equipment

**Fig 4:**
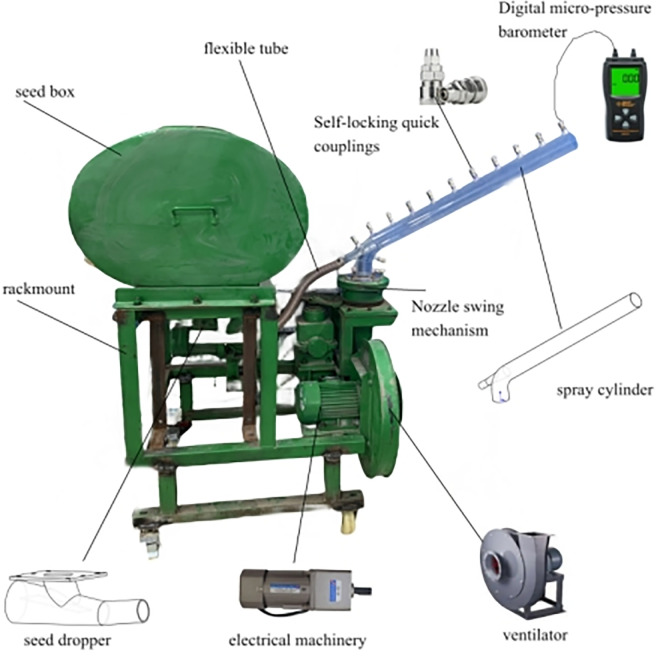
Experimental equipment.

Molding pneumatic seeder: The 300W speed-regulating motor powers the fan via frequency modulation. The reducer controls the seed falling speed [21]. The 9–19-4A high-pressure centrifugal fan generates high-speed airflow. The C-type self-locking quick connector measures pressure at a fixed monitoring point with a barometer, while the handheld micro pressure barometer (0–10000 Pa, 1 Pa resolution) measures and reads the pressure at the monitoring point. Fig 4 shows the experimental equipment.

**Fig 5:**
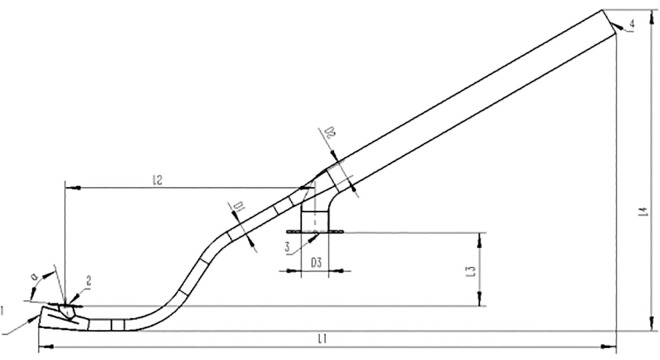
Pneumatic convey system 2D dimensional drawing. 1. Seed drop air inlet 2. Seed box connection port 3. High-speed fan connection 4. Air outlet slit.

Fig 5 illustrates the design dimensions of the pneumatic conveying system, while Table 1 details its operating parameters. The geometric parameters include the inclination angle α between the seed dropper and the horizontal plane, the throat’s outlet diameter *D*_2_, and the inlet diameter *D*_1_. Additional specifications are a nozzle diameter of 76 mm, a tube wall thickness of 1 mm, a vertical distance of 205 mm between the seed inlet and the high-pressure blower connection port, and a distance of 704 mm between the seed dropper’s inlet center and the nozzle blower’s inlet center. The throat diameter is 31 mm, with a total system length of 1628 mm and an overall height of 905 mm.

**Table 1:** Geometric parameter.

Parameter code	Parameter description	numeric size
α	Seed dropper seed inlet inclination (°)	70
*D*_1_	Diameter of the throat inlet (mm)	31
D_2_	Diameter of the throat outlet (mm)	50
*D*3	Fan inlet diameter (mm)	76
*L*_1_	Total length of pneumatic convey system (mm)	1628
*L*_2_	Horizontal distance between seed inlet and fan inlet (mm)	704
*L*_3_	Vertical distance between seed inlet and fan inlet (mm)	205
*L*_4_	Pneumatic convey system full height (mm)	905

**Fig 6:**
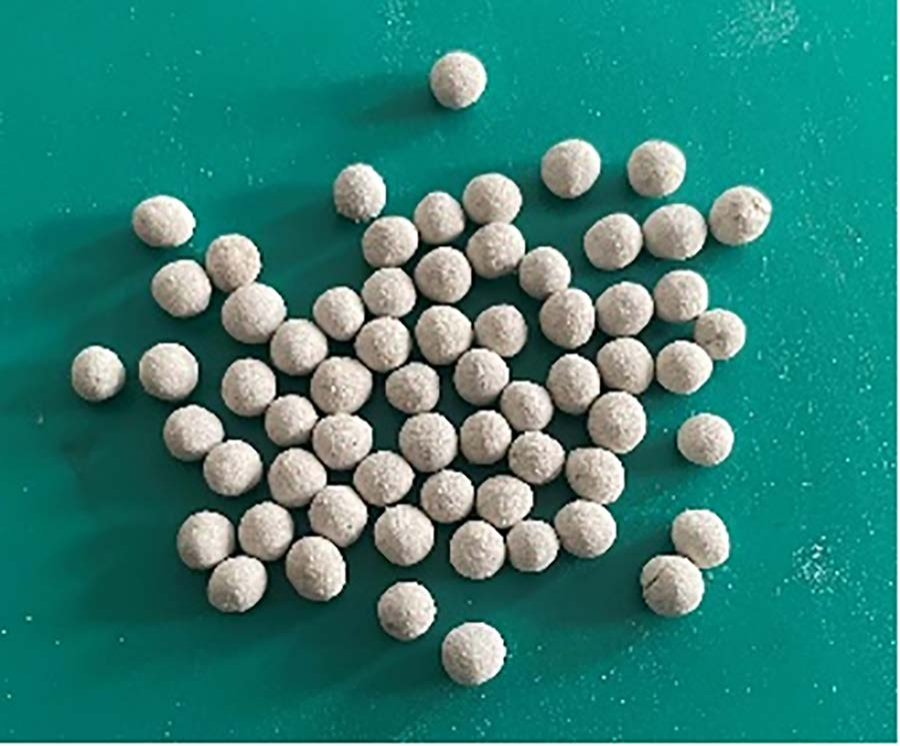
Coated seeds.

The experimental material used in this test is coated seeds, as depicted in Fig 6. These seeds have the following parameters: a density of 1279 ± 0.035 kg/m³, with overall dimensions of 2.0 ± 0.125 mm in length, 1.5 ± 0.054 mm in width, and 1.1 ± 0.062 mm in height.

### Experimental procedures

**Fig 7:**
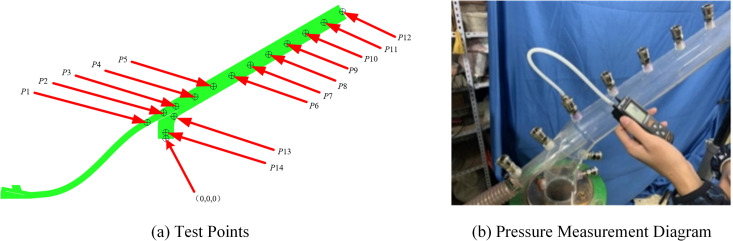
Arrangement of pressure sensors and physical drawings.

The experiment began with the application of a protective layer to the seeds. To measure the pressure distribution inside the nozzle at different inlet air speeds, 14 equidistant points were selected along the nozzle and perforated, labeled *P*_1_, *P*_2_, *P*3,..., *P*_14_, as shown in the simulation model diagram. Pressure measuring heads were installed at each perforation, as depicted in Fig 7. During the experimental phase, fan frequency levels were progressively increased, and a handheld digital micro-pressure gauge was used to measure and record the pressure values at each designated point. To minimise experimental error, each test was repeated three times, and the average value was calculated for analysis. Resulting in the final test data presented in Table 2.

**Table 2:** Coordinates of measurement points.

Point	X/ (mm)	Y/(mm)
*P*_1_	−89	77
*P*_2_	−10	121
*P*_3_	50	147
*P*_4_	136	196
*P*_5_	223	246
*P*_6_	309	296
*P*_7_	396	346
*P*_8_	483	396
*P*_9_	569	446
*P*_10_	656	496
*P*_11_	743	546
*P*_12_	830	596
*P*_13_	38	105
*P*_14_	0	30

## CFD simulation

### Fluid mechanics mode

The pneumatic seeder operates under rarefied phase flow conditions, where CFD simulations are employed to analyze the velocity and pressure distribution within the flow field, as well as the kinematic behavior of seeds. In this context, the Eulerian-Lagrangian approach is applied. For an incompressible Newtonian fluid, the primary governing equations of fluid motion include the continuity equation for mass conservation and the momentum equation, commonly referred to as the Navier-Stokes (NS) equations [26–28].

Mass conservation equations:


$$\frac{\partial v{\mathrm{\backslash nolimits}}_{X}}{\partial x}+\frac{\partial v{\mathrm{\backslash nolimits}}_{y}}{\partial y}+\frac{\partial v{\mathrm{\backslash nolimits}}_{z}}{\partial z}=0$$ (1)

Introduction of Hamiltonian differential operators [29]:


$$\nabla =i\frac{\partial }{\partial x}+j\frac{\partial }{\partial y}+k\frac{\partial }{\partial z}$$ (2)

Then equation 1 is written:


$$\frac{\partial \rho }{\partial t}+\nabla \cdot (\rho V_{X})=0$$ (3)

Momentum equation:


$$\frac{\partial V}{\partial t}+V.\nabla V=-\frac{\text{1}}{\rho {\mathrm{\backslash nolimits}}_{g}}\nabla p+F{\mathrm{\backslash nolimits}}_{volume}+v\nabla {\mathrm{\backslash nolimits}}^{2}V$$ (4)

*V*_x_, *V*_y_, and *V*_z_ represent the velocity components in the *x*, *y*, and *z* directions respectively; The unit vectors *i*, *j*, and *k* correspond to the *x*, *y*, and *z* axes in three-dimensional coordinates; *P* denotes gas pressure while *F* body represents the volume force exerted by gravity on particles interacting with airflow; *ρ*_g_ signifies air density.

According to the fundamental principles of fluid mechanics, the Reynolds number is a crucial criterion for distinguishing the flow characteristics of fluids. When the Reynolds number exceeds the critical threshold in a given flow field, turbulent flow is observed; When it equals the critical value, critical flow occurs; And when it falls below this threshold, laminar flow prevails [30–32].

Reynolds Number Equation:


$$Re\mathrm{\backslash nolimits}=\frac{vd}{\mu }$$ (5)

*v* is the average flow in the flow field; *d* is the throat diameter; *μ* is the kinematic viscosity of air.

To ensure that the overall model is properly closed, the port on the seed dropper connected to the seed box and the port open to the atmosphere are set to pressure inlets, with the hydrostatic pressure set to atmospheric pressure. Similarly, the outlet of the pneumatic seeder canister is defined as a pressure outlet, also set to atmospheric pressure.

The flow field within the pneumatic convey system is characterized by strong turbulence, making the gas-phase flow turbulent in the gas-solid two-phase flow. To analyze the internal fluid dynamics of the seed discharge system, a turbulence model is employed. The high-speed jet generated by the sprayer nozzle involves complex flow phenomena, including boundary layer separation, particle–airflow interaction, and flow diversion around blunt bodies, the RNG k-ε model based on Reynolds-averaged Navier–Stokes (RANS) equations offers an effective compromise between computational cost and simulation accuracy, The RNG k-ε model enhances prediction accuracy in high-shear regions of high-speed jets, such as the core decay zone, by refining the dissipation rate equation of turbulent kinetic energy. However, its assumption of isotropic eddy viscosity may lead to underestimation of turbulence modulation in regions with intense particle–flow interactions, potentially causing deviations in jet spreading and particle distribution predictions. Meanwhile, empirical settings of inlet turbulence parameters (e.g., turbulence intensity, viscosity ratio), sensitivity to near-wall mesh resolution, and omission of particle-phase turbulence modulation, which requires coupling with a DPM model, introduce uncertainties. These must be addressed through experimental calibration of jet velocity and particle concentration fields, supplemented by refined boundary-layer meshing and mesh-independence validation to ensure simulation reliability.

### Geometric model

**Fig 8:**
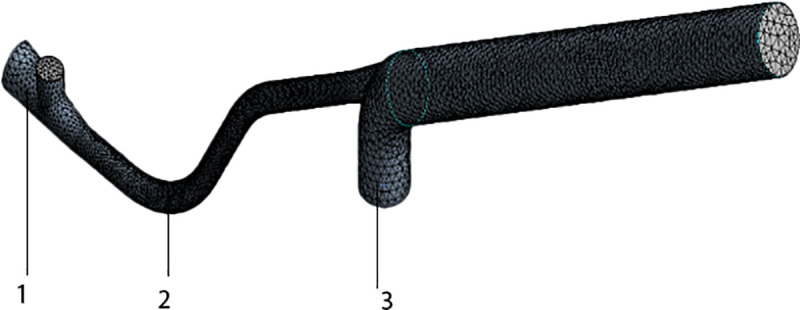
Grid division of pneumatic seeder. 1. pneumatic seeder 2. throat 3. nozzle.

We analyze the gas-solid two-phase flow field of the original prototype pneumatic conveying system. To simplify the model, this study focuses on the section above the swinging disc. Fig 8 presents a two-dimensional schematic diagram of the nozzle in the pneumatic conveying system, which consists of three main components: the pneumatic seeder, throat, and nozzle. The nozzle is connected to the throat, with the other end of the throat linked to the pneumatic seeder. The outlet of the nozzle and the left end of the pneumatic seeder are open to the atmosphere, while the upper interface of the pneumatic seeder is connected to the seed box. Additionally, the lower interface of the nozzle is connected to a fan.

Firstly, three-dimensional models of a pneumatic seeder, plastic throat, and nozzle were constructed and assembled by using SolidWorks. Then, these models were imported into SpaceClaim to extract the fluid domain, which was subsequently imported into Workbench for grid division and meshing.

The meshing process in Workbench was carried out with the following settings and parameters: a unit size of 93.156 mm, a growth rate of 1.2, a maximum size of 186.31 mm, a feature clearance size of 0.465 mm, and a minimum curvature size of 0.931 mm. The normal curvature angle was set at 18°, with a boundary box diagonal of 1864.6 mm and an average surface area of 15,226 mm². The minimum edge length was 1.5004 mm, and the target skewness was 0.9. A medium smoothness level with a transition ratio of 0.272 was applied, with a maximum of five layers.

In the meshing process, the sweepable portions of the model were subjected to tetrahedral mesh generation, while the non-sweepable portions were handled with hexahedral mesh generation. Additionally, a mesh sensitivity analysis was conducted through an irrelevance test to ensure that the mesh quality was sufficient for accurate simulation results.

In Fig 8, the first inlet is designated as “inlet1” and configured as a pressure inlet. The second inlet, labeled “inlet2”, is also set as a pressure inlet. The third inlet, named “inlet3”, functions as a velocity inlet with an initial velocity of 10 m/s, which increases gradually to 32.5 m/s. The fourth inlet is identified as “outlet,” and the throat wall is defined as a no-slip wall, referred to as “wall”.

### Grid-independent verification

The mesh resolution influences the accuracy of computational results. Therefore, a mesh-independence test is essential to determine the optimal mesh density. The computational domain model is subsequently subjected to meshing for this purpose. Excessively coarse grids may result in inaccurate or non-convergent solutions, while overly fine grids significantly increase computational time. To ensure both accuracy and efficiency, five grid sizes will be compared and analysed, as detailed in Table 3.

**Table 3:** Grid division dimensions.

Mesh case	Mesh size	Grid number
1	360	100997
2	180	186322
3	93	361991
4	70	470943
5	30	624620

To clarify the influence of grid resolution on simulation accuracy, deviation calculations were performed across different grid sizes, using the following formula:


$$Deviation\;=\frac{Outlet\;velocity\;for\;a\;given\;size\;grid-Average\;outlet\;velocity\;for\;five\;grids}{Average\;outlet\;velocity\;for\;five\;grids}\times 100\%$$ (6)

**Fig 9:**
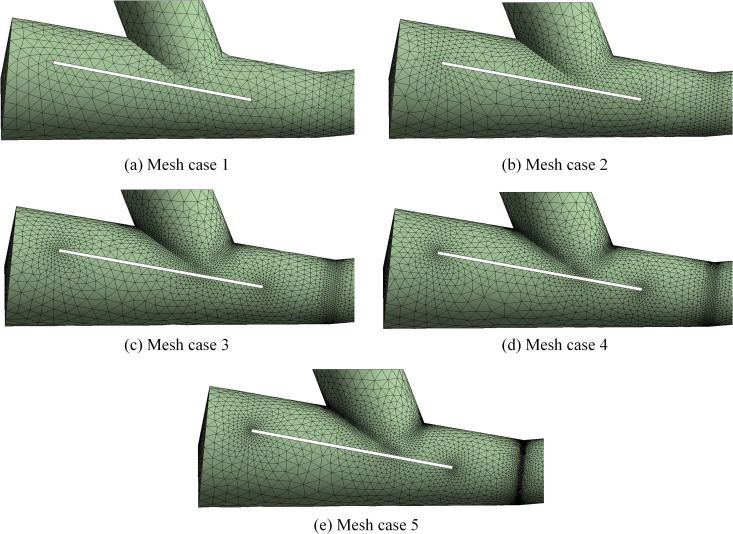
Detailed mesh distributions corresponding to five different grid resolutions.

Fig 9 presents the mesh distributions corresponding to the five grid sizes. The detailed view reveals substantial differences in mesh density across the grid levels, meeting the requirements for grid-independence verification.

**Fig 10:**
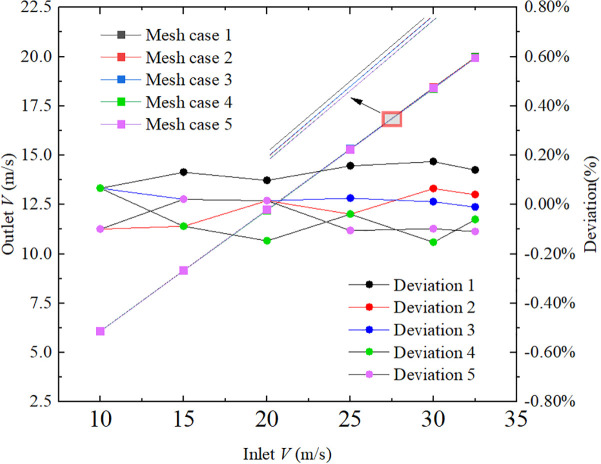
Grid independence verification.

Flow field simulations were conducted using five different mesh configurations and six inlet velocity conditions for “inlet3” (10 m/s, 15 m/s, 20 m/s, 25 m/s, 30 m/s, and 32.5 m/s), with outlet velocity as the output variable. All other parameters were held constant, and the corresponding results are presented in Fig 10.

As shown in the figure, variations in grid quantity exert minimal influence on the simulation outcomes. Balancing computational accuracy and efficiency, a grid size of 93 mm with 361,991 cells was selected for subsequent analyses, meeting the requirements of grid independence.

### Comparison of simulation and bench test results

**Fig 11:**
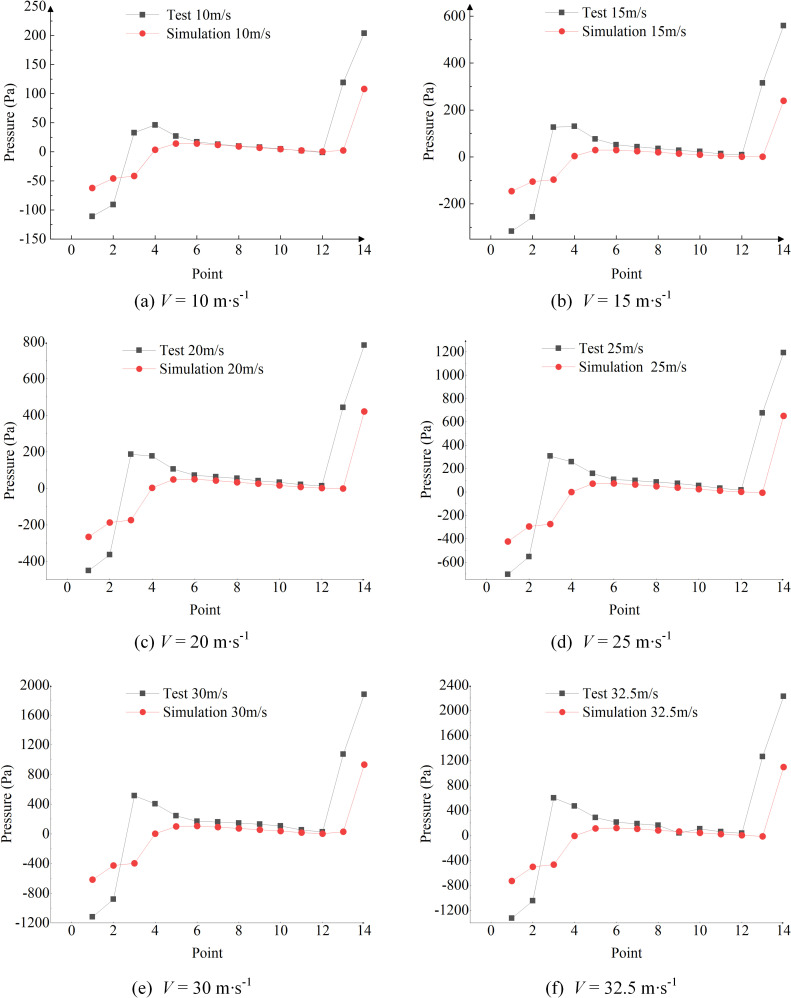
Experimental results and simulation data.

The test and simulation data were plotted to show the corresponding speeds. A comparison graph was then created to illustrate the pressure at six wind speeds (10 m/s, 15 m/s, 20 m/s, 25 m/s, 30 m/s, and 32.5 m/s). Fig 11 presents the comparative relationship between the experimental results and the corresponding simulation data.

**Fig 12:**
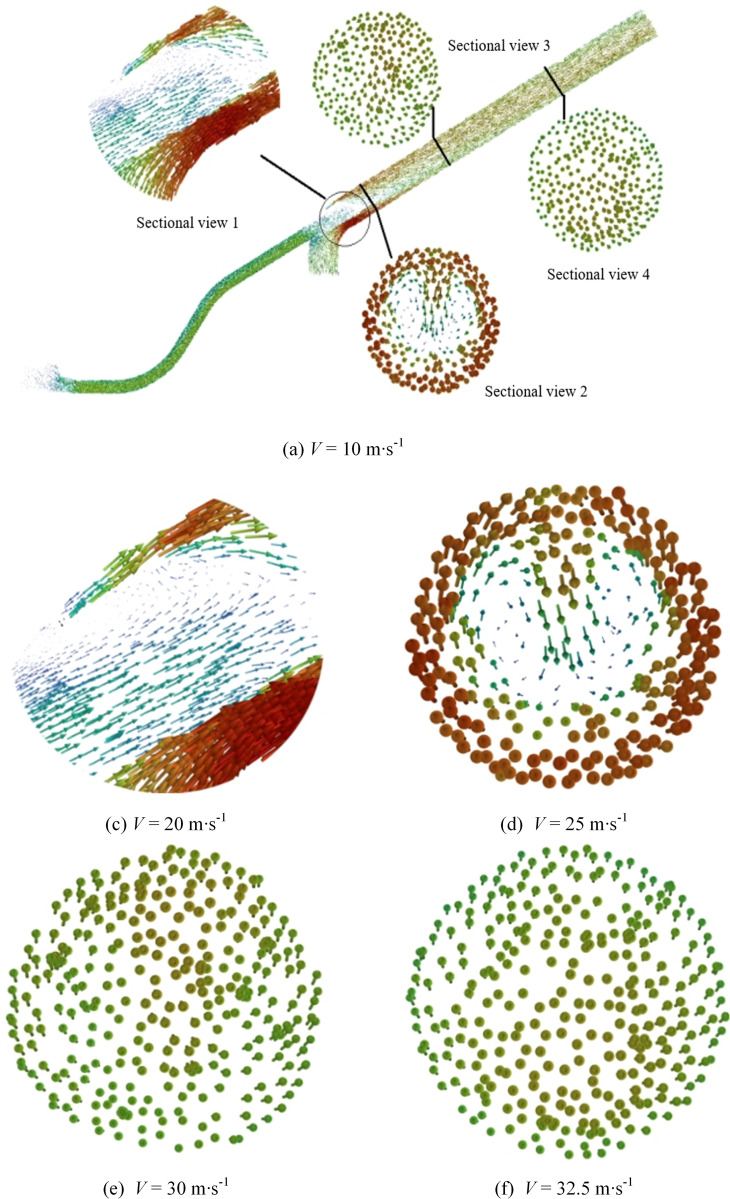
Velocity clouds and magnified views of the local spray flow field.

Comparing the simulated and tested pressures at various wind speeds reveals a general consistency between the results, though some discrepancies are evident. Due to the structural characteristics of the system, the vortex formation at the throat outlet creates specific phenomena: high-speed airflow is expelled through the nozzle, while a secondary flow generated by negative pressure at the throat outlet enters the main airflow with lower velocity. The locations of these eddy currents are illustrated in Fig 12, with enlarged and partially enlarged diagrams showing their shape, trend, and intensity. Despite slight deviations between the measured pressures (*P*_1_ to *P*_4_) and the ideal pressure data obtained through simulation. The comparison between the simulated and tested pressure data at various wind speeds shows a general consistency. The broken line graph illustrates a consistent and ideal simulated data, the overall trend shows a consistent upward pressure at points *P*_1_ to *P*_3_ and approaches atmospheric pressure at points *P*_4_ to *P*_12_, and the pressure approaches atmospheric pressure in both theory and practice. Points *P*_13_ and *P*_14_ exhibit an increasing pressure trend. These findings suggest that while random measurement errors and vortex formation introduce some discrepancies, simulations can effectively guide improvements in pneumatic seeder performance.

### Optimized design

Based on the working principle of the pneumatic seeder and an analysis of fluid dynamics, eddy currents frequently develop at the throat outlet, which can lead to blockages in the pneumatic conveyance system. Testing has also demonstrated that seed breakage commonly occurs at the nozzle outlet. To address these issues, a control variable analysis was conducted based on the original prototype’s structural parameters (*D*_1_ = 31 mm, *D*_2_ = 50 mm, *α* = 70 °), aiming to evaluate the impact of varying each parameter on spraying performance relative to the prototype, thereby identifying the optimal structural configuration. The study was conducted in three stages: firstly, optimizing the throat outlet diameter; secondly, optimizing the throat inlet diameter; and finally, analyzing the influence of different seed inlet inclination angles on seed breakage within the conveying system. Discrete phase modeling (DPM) was employed to analyze the effects of various seed entrance angles on particle-wall collisions. Key variables, such as jet source particle diameter, surface area, and total mass flow rate, were adjusted to visualize collision dynamics through trajectory maps. These adjustments aimed to optimize seed-wall interactions and minimize seed crushing. Initial modifications focused on adjusting the throat tube diameters, followed by changes in the seeder’s tilt angles while maintaining other parameters constant. This comprehensive approach provided a refined analysis of each variable’s impact on collision speed, ultimately enhancing the performance of pneumatic seeders.

In the simulation, to ensure the pneumatic seeder operates effectively at an airflow velocity of 32.5 m/s without excessive jitter and while preserving its service life, it is recommended that the initial airflow velocity into the fan be kept below 32.5 m/s. Although higher initial airflow velocities can reduce the likelihood of blockages by generating greater negative pressure and increased speed, they also heighten the risk of damaging the seeder. Accordingly, the inlet air velocity in the simulation was defined as 30 m/s to reflect the actual operating conditions.

### Effect of export diameter on pneumatic convey system of pneumatic seeders

Based on the analysis of the pneumatic seeder’s working principle, eddy currents are expected to form at the top of the throat outlet, aligned with the nozzle. Therefore, optimizing the throat diameter is essential for minimizing these currents and improving performance. By maintaining a constant inlet diameter and tilt angle of the throat in the pneumatic conveyor system, the outlet diameter was varied for simulation analysis. The effects of throat outlet diameters of 40 mm, 45 mm, 55 mm, and 60 mm on air velocity were investigated and compared with the simulation results of the original pneumatic planter (*D*_2_ = 50 mm), to determine the optimal throat outlet diameter for achieving maximum air velocity. The throat structure was modified, and corresponding simulation models were developed to calculate and evaluate airflow field velocity parameters. To ensure accurate comparisons, consistent boundary conditions and simulation environments were maintained across all models, with throat tube dimensions as the only variable. This study aims to assess the influence of throat diameter on airflow dynamics at this critical location.

**Fig 13:**
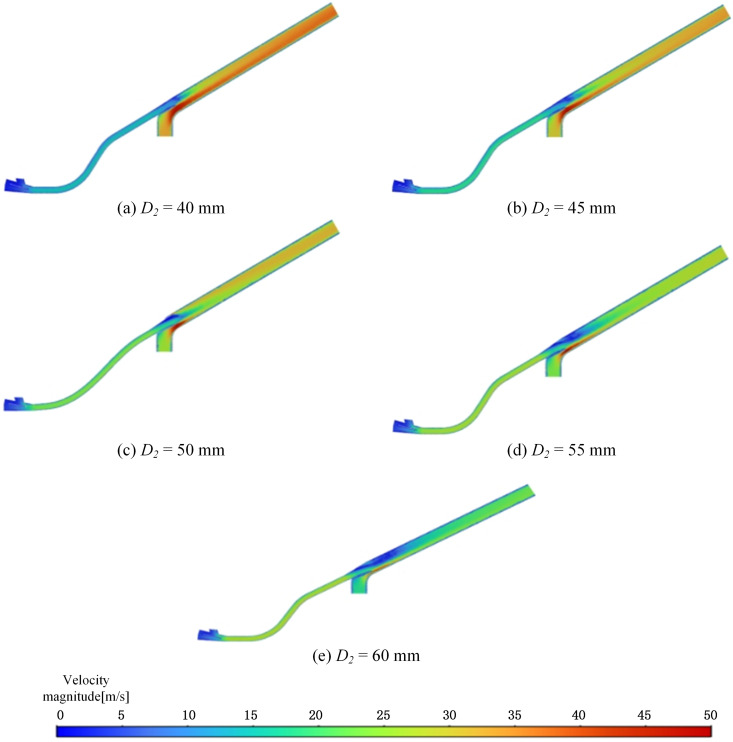
Velocity clouds at each diameter.

Analysis of the velocity mapping results following the optimization of the throat’s structural parameters reveals key insights. After configuring the parameters in Fluent and running the calculations, the resulting velocity contour maps for each parameter are presented in Fig 13.

The velocity field distribution results indicate that the airflow velocity near point *P*_3_ at the center of the throat varies with the throat diameter. Specifically, at *D*_2_ = 40 mm, the velocity ranges from 9 to 14 m/s; At *D*_2_ = 45 mm, the velocity increases to 10–15 m/s; At *D*_2_ = 55 mm, the velocity peaks at 18–26 m/s; At *D*_2_ = 60 mm, the velocity decreases to 8–16 m/s. The results indicate that the velocity at the throat outlet generally increases with an increase in diameter. However, when *D*_2_ = 55 mm, a further increase in diameter leads to a decrease in airflow velocity. Based on these findings, it can be concluded that altering the diameter of the throat outlet is more effective in reducing vortices and mitigating the risk of clogging in pneumatic conveying systems. The velocities at *P*3 are summarized for various tube outlet diameters in Table 4.

**Table 4:** Velocity under different diameters at *P*3.

*D*_2_ (mm)	40	45	50	55	60
*V* (m·s^-1^)	9.6	11.9	15.8	18.4	13.5

**Fig 14:**
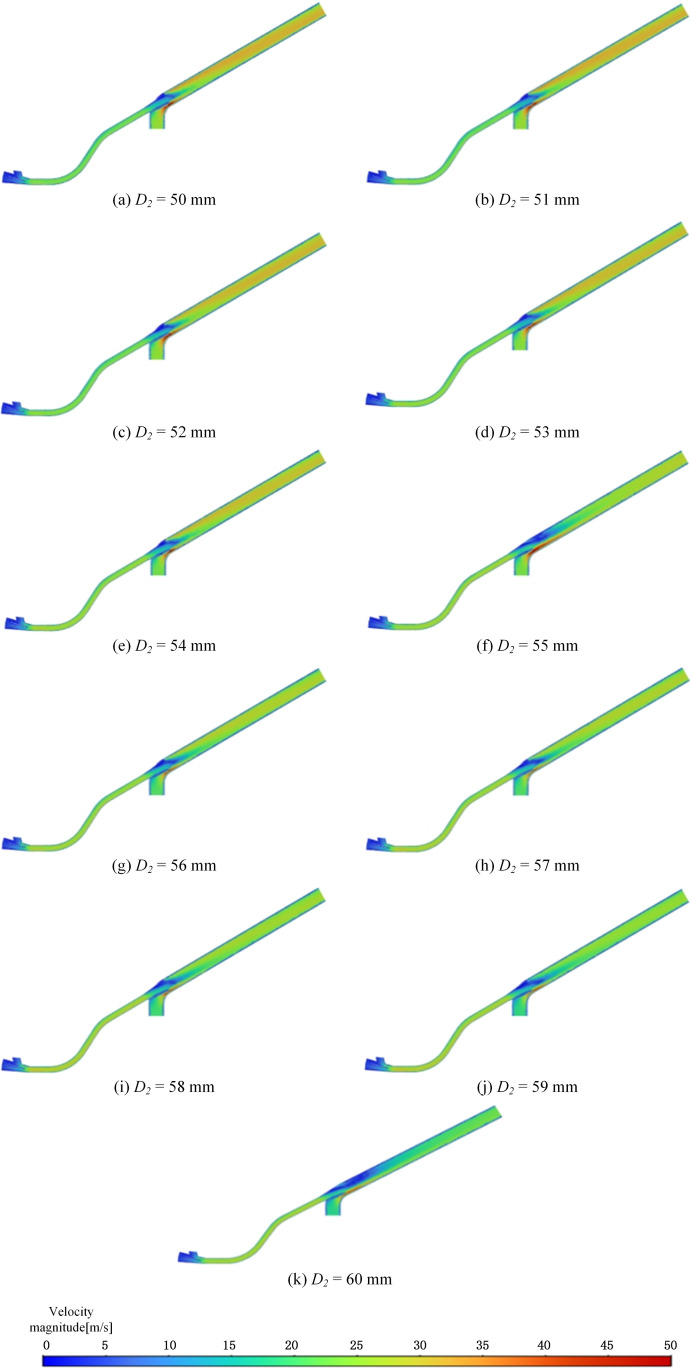
Velocity clouds with refined parameters.

The velocity data at point *P*_3_ confirms that the velocity at this location aligns with the ranges shown in the velocity cloud chart. As the throat diameter increases, the velocity at the center of the throat outlet also increases, but after reaching a certain diameter, the velocity begins to decrease. This makes it difficult to pinpoint the exact optimal diameter. Therefore, further refinement of the structural parameters is necessary to identify the optimal configuration that minimizes eddy currents and enhances seed transportation. The simulation results for these refined parameters are illustrated in Fig 14

**Fig 15:**
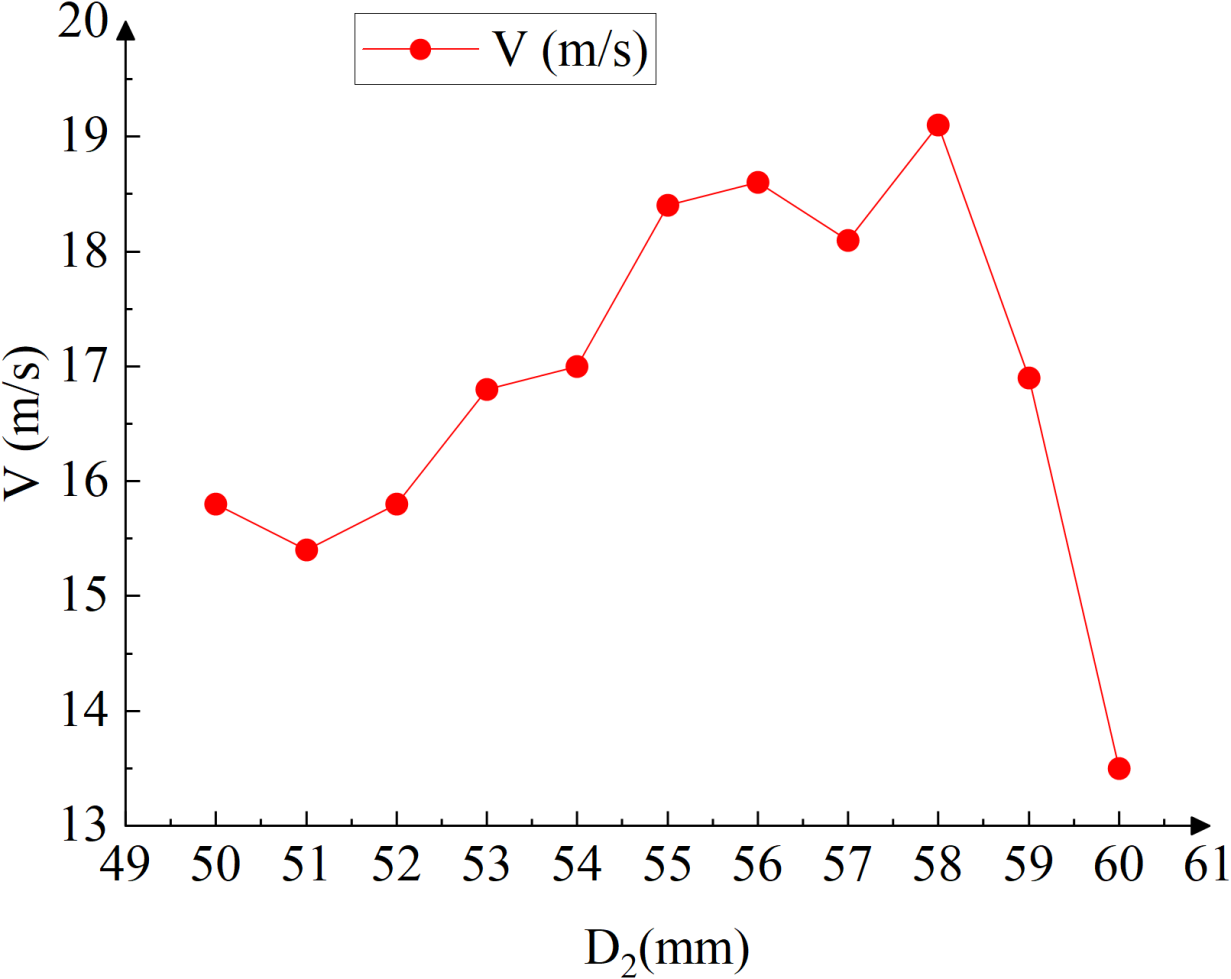
Velocity under different diameters at *D*_2_.

The analysis of the velocity ranges in the cloud chart reveals that when the diameter is between 50–54 mm, the velocity at the head of the pneumatic seeder barrel is higher compared to diameters of 55–60 mm. However, this trend does not hold at the throat of the pneumatic seeder barrel due to the presence of eddy currents at the throat outlet, which disrupts the expected fluid dynamics. Despite this anomaly, the optimal velocity can still be identified based on the cloud chart results. Comparison of Fig 14a and 14i, after optimising the throat outlet diameter, the velocity distribution within the structural transition zone becomes more uniform, and regions of locally high velocity gradient are significantly attenuated. Notably, the velocity discontinuity and recirculation zones at the junction between the throat outlet and the main conveying pipe are effectively suppressed. The contraction effect of the fluid passing through the throat is mitigated, reducing the velocity gradient and significantly inhibiting vortex formation induced by abrupt geometric transitions, thereby enhancing flow field stability; Meanwhile, the smoother geometric transition mitigates energy loss caused by high-speed flow impingement on the wall, thereby reducing system pressure loss and enhancing overall conveying efficiency. A more stable flow field also minimizes flow-induced disturbances on seeds during transport, effectively decreasing seed-wall collision frequency and lowering the risk of fragmentation and clogging. Therefore, the optimisation of these structural parameters is considered highly beneficial, as it enhances seed delivery performance while markedly reducing the likelihood of seed breakage. In this study, the velocity at point P_3_, located at the throat outlet, was used as a key indicator. The velocities at P_3_ under different structural configurations are summarized in Table 5 and illustrated in Fig 15.

**Table 5:** Velocity under different diameters at *P*3.

*D*_*2*_ (mm)	50	51	52	53	54	55	56	57	58	59	60
*V* (m·s^-1^)	15.8	15.4	15.8	16.8	17	18.4	18.6	18.1	19.1	16.9	13.5

Based on the velocities measured at point *P*_3_, it can be concluded that the maximum outlet velocity occurs when *D*_2_ = 58 mm, the air velocity peaks at 19.1 m/s, minimizing the likelihood of vortex formation, representing a 20% increase compared to when *D*_2_ = 50 mm. Optimizing the throat outlet diameter effectively reduces eddy currents, playing a crucial role in mitigating the risk of clogging in pneumatic conveying systems. Therefore, a throat diameter of 58 mm is identified as the critical parameter for ensuring optimal and unobstructed operation in pneumatic conveying systems.

### Effect of the diameter of the throat inlet on pneumatic convey system of aerial pneumatic seeder

**Fig 16:**
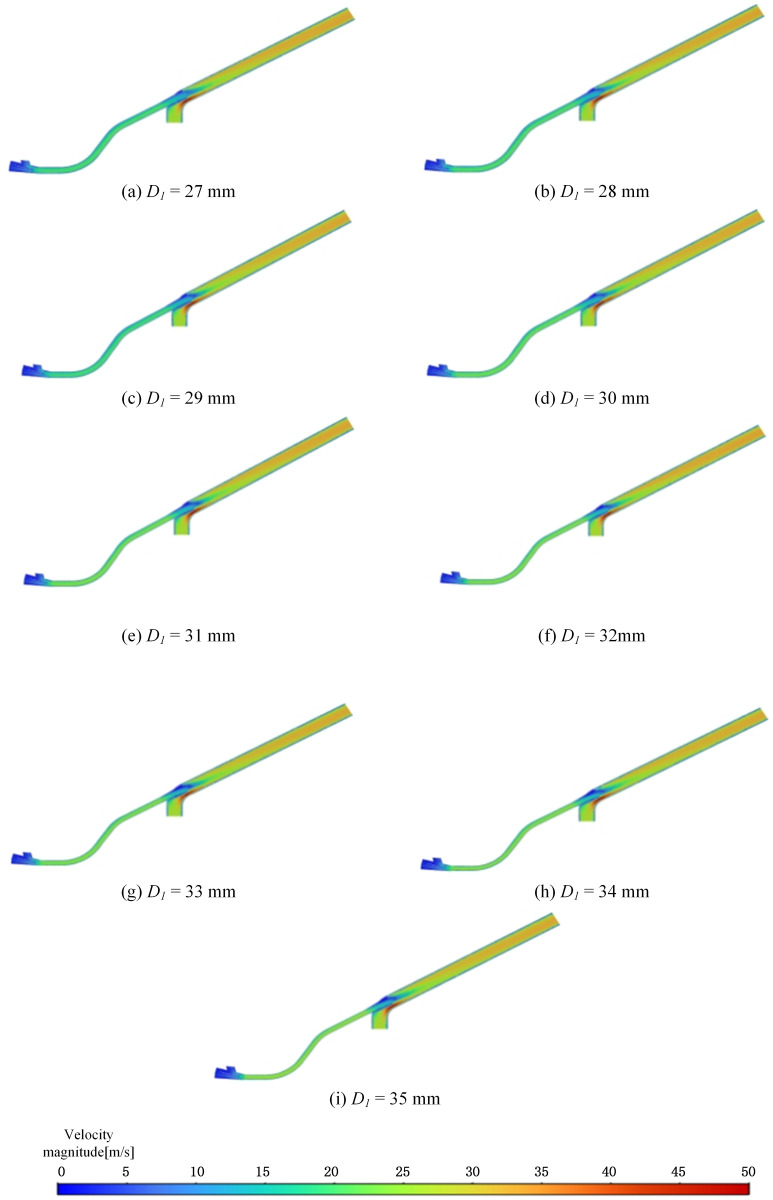
Optimized speed cloud of reduction ratio parameters.

In this section, the throat outlet diameter and tilt angle of the pneumatic conveyor system are kept constant, while the throat inlet diameter is varied in the simulation analysis to identify the optimal diameter that enhances system performance without compromising normal operation. The impact of seed throat inlet diameters of 27 mm, 28 mm, 29 mm, 30 mm, 32 mm, 33 mm, 34 mm, and 35 mm on airflow velocity was analyzed and compared with the simulation results of the original pneumatic seeder (*D*_1_ = 31 mm) to determine the optimal throat inlet diameter for maximizing airflow velocity. After configuring the parameters in Fluent, the velocity distribution results following the optimization of the throat inlet diameter are presented in Fig 16.

The results from the velocity cloud charts indicate that the velocity at the throat outlet remains relatively low, while velocities increase as the flow approaches the atmospheric pressure outlet in the nozzle. The maximum velocity is observed at the connection between the fan and the area below the throat. These general trends are consistent across different configurations. However, the variations in velocity within the throat, throat, and nozzle sections are minimal, as evidenced by similar color distributions in the cloud charts under various parameter conditions. This indicates that variations in the throat inlet diameter have a minimal impact on airflow velocity. Comparison of Fig 16b and 16e, the comparison of velocity contours before and after the throat inlet diameter optimisation indicates that the velocity gradient remains largely unchanged, and the reflux phenomenon at the throat outlet is not effectively suppressed. Therefore, this structural optimisation yields limited improvement and is deemed to have marginal significance. The velocity at point *P*_3_ continues to be used as the evaluation metric, with the detailed values provided in Table 6.

**Table 6:** *P*3 point speed.

*D*_*1*_(mm)	27	28	29	30	31	32	33	34	35
*V*(m·s^-1^)	15.5	16.8	16.2	15.9	15.8	16.1	15.9	15.9	15.6

Due to the constraints of the machine’s structure, the range of variation for the throat inlet diameter is limited. The air velocity results at point *P*3 demonstrate that changes in the throat inlet diameter have a negligible impact on airflow velocity. When *D*_1_ = 28 mm, the maximum velocity reaches 16.8 m/s, representing only a 6% increase compared to the 15.8 m/s observed at *D*_1_ = 31 mm. Thus, optimizing the throat inlet diameter has little impact on reducing vortices and does not notably decrease the risk of clogging in pneumatic conveying systems.

### Effect of tilt angle on pneumatic convey system of pneumatic seeder

In this section of the study, the tilt angle of the pneumatic conveyor system is varied while keeping the throat inlet and outlet diameters constant. The simulation and analysis aim to determine the optimal tilt angle that enhances system performance without disrupting normal operation. The seed-wall collision frequency at tilt angles of 60°, 65°, 75°, and 80° was examined and compared to that of the original pneumatic planter with a tilt angle (*α* = 70°). A discrete phase model (DPM) was employed to simulate seed-wall collisions following modifications to the seed inlet structure, and a corresponding model was developed to evaluate the resulting impact. The particle diameter was set uniformly at 3 mm, with the total mass flow rate maintained between 10–20 kg/s. The particles were then introduced into the pellet system. This configuration effectively analyzes the impact of tilt angle variations on seed-wall collisions and identifies the optimal tilt angle to minimize such collisions.

**Fig 17:**
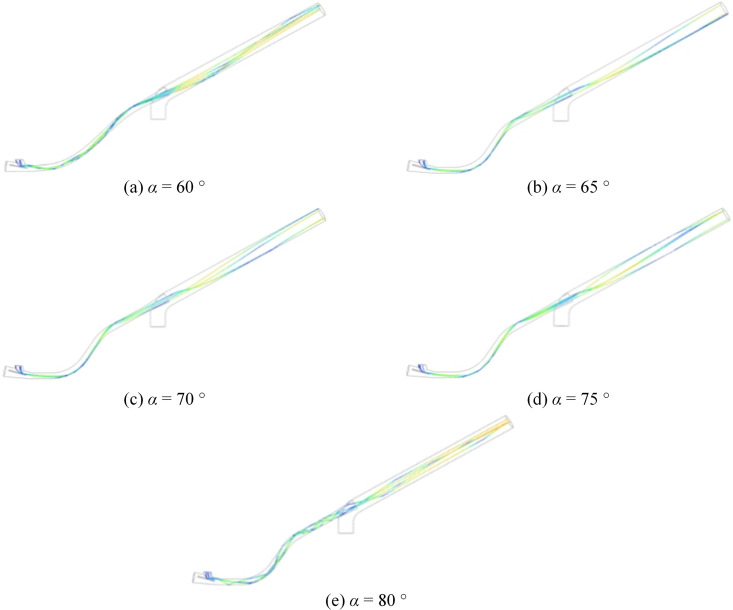
Particle and wall collision diagram.

The particle direction collision trajectory diagram was created by setting up the Discrete Phase Model (DPM) with relevant particle direction parameters, analyzing the particle trajectories and their collision patterns with the tube wall. The resulting trajectory graph is displayed in Fig 17.

**Fig 18:**
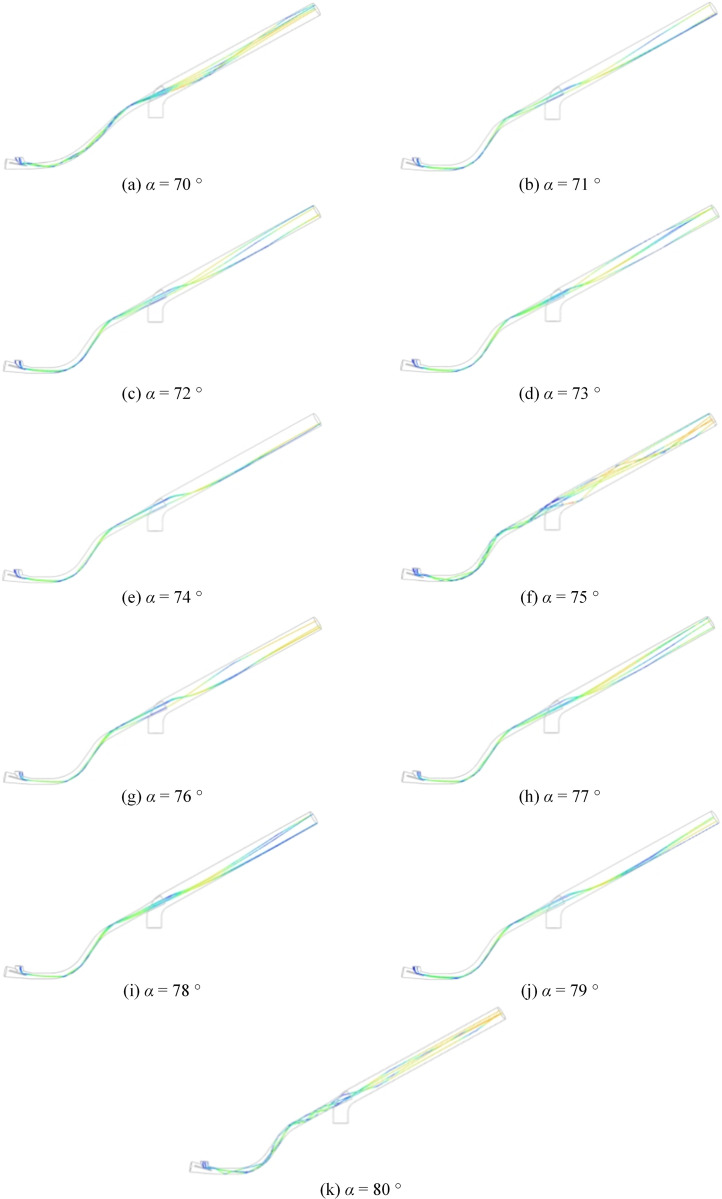
Collision refinement parameter.

According to the trajectory velocity diagram of particle directions, it is evident that the majority of collisions occur within the throat. Collisions in the throat result in a slight reduction and subsequent increase in velocity, with a relatively small difference before and after collision. Conversely, collisions within the pneumatic seeder barrel also led to deceleration followed by acceleration. However, the disparity between velocities before and after collision is greater than that observed in the throat. Moreover, overall velocity within the nozzle surpasses that within the throat. Table 7 illustrates the relationship between particle-wall collisions and inclination angles. As the seed inlet angle increases, the total number of collisions initially decreases; however, when the angle exceeds *α* = 75°, the collision frequency begins to rise. Therefore, optimizing the seed inlet angle can significantly reduce the total number of particle-wall collisions.. Nevertheless, it remains uncertain whether there exists an optimal parameter value between 70° and 80°. Therefore, further subdivision of tilt angles is necessary, ensuring a progressive increase in subdivision angles. The result is shown in Fig 18.

**Table 7:** Collision times after parameter optimization.

tilt angle (*α*/°)	60	65	70	75	80
Number of *collisions*	12	12	10	6	12

Comparison of Fig 18a and h, the comparison of particle flow contours before and after optimising the seed incidence tilt angle reveals that the adjusted trajectory promotes the formation of a stable Coriolis-induced secondary flow, resulting in a helical transport flow field. The established flow regime generates a “fluid cushion” effect via fluid-solid interaction, transitioning seed movement from discrete, high-energy collisions to continuous, quasi-static dense packing, this shift lowers the impact energy density below the elastic deformation threshold of the seed coat, thereby markedly reducing the likelihood of seed damage; Meanwhile, adjusting the incidence angle restructures the momentum transfer topology between the seed flow and boundary surfaces, shifting the dominant collision mechanism from normal impact to tangential slip. This geometric optimisation of momentum dissipation pathways effectively reduces the risk of seed fragmentation. Therefore, the optimisation of these structural parameters plays a crucial role in effectively reducing the likelihood of seed breakage.

**Fig 19:**
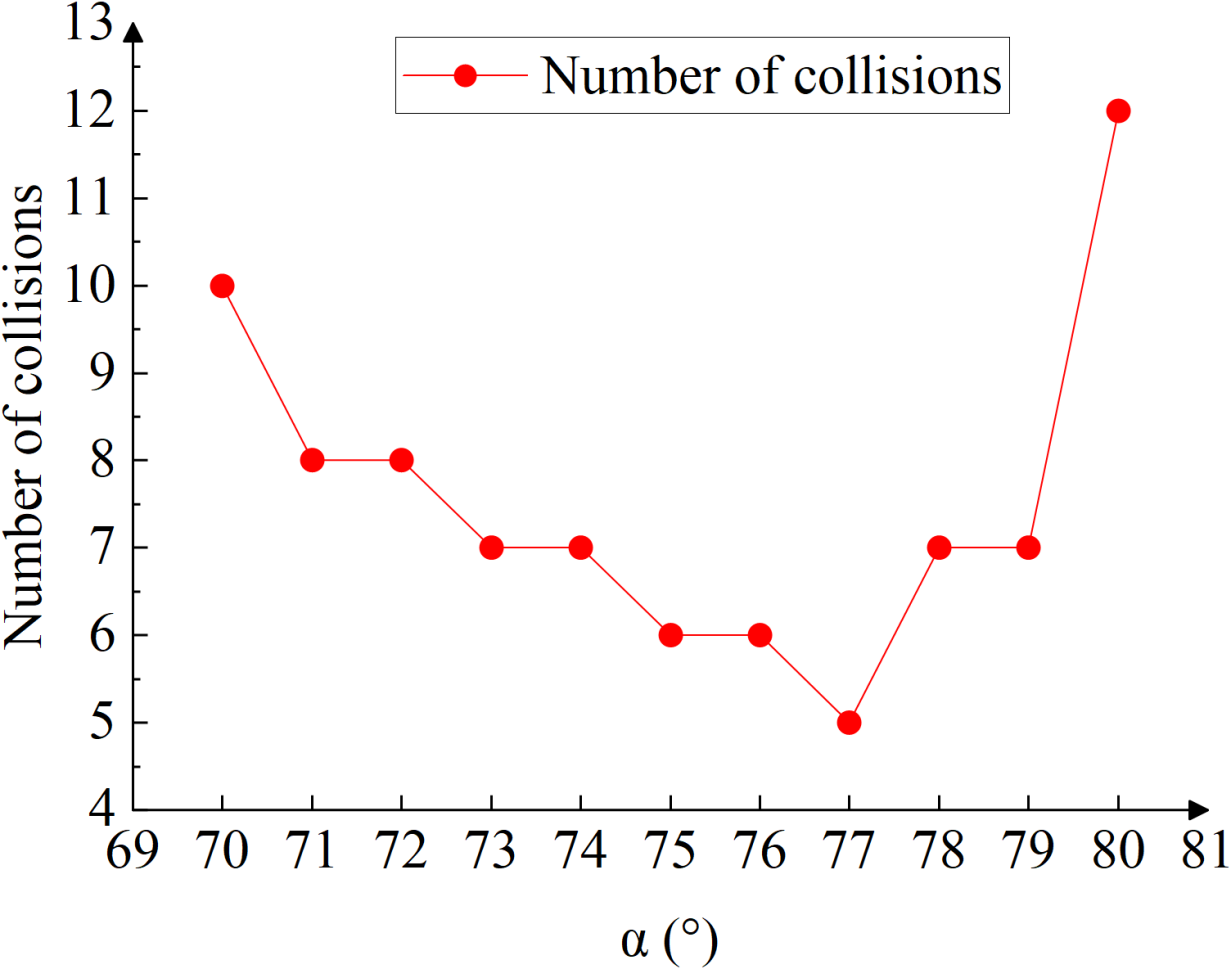
Number of collisions at each angle.

The particle collision trajectory and the corresponding velocity change trend exhibit a pattern consistent with the particle collision trajectory observed before subdivision. By analyzing the particle-wall collision trajectories shown in Fig 19, the average number of collisions can be calculated. The results of these collision counts are presented in the Table 8.

**Table 8:** Number of collisions at each angle.

tilt angle (*α*/°)	70	71	72	73	74	75	76	77	78	79	80
Number of collisions	10	8	8	7	7	6	6	5	7	7	12

Based on the trend shown in Fig 19, it can be concluded that the collision frequency reaches its minimum value of 5 collisions when the seed drop device tilt angle is *α* = 77 °. The collision probability is reduced by 50% compared to *α* = 70 °. Therefore, optimizing the seed dropper inlet tilt angle can significantly decrease the likelihood of seed breakage, which is crucial for maintaining seed integrity during pneumatic conveying.

### A comparative analysis of the simulation results conducted before and after the model optimization

Table 9 presents a comparative analysis of the optimised throat inlet diameter, throat outlet diameter, and seed incidence tilt angle.

**Table 9:** Comparison of original and optimised structural parameters.

Parameter code	original size	Optimised size	Single-item optimisation effects
*D1*	31	28	20% speed increase at *P*3
*D*_2_	50	58	6% speed increase at *P*3
*α*	70	77	Reduce the probability of seed breakage by 50%

**Fig 20:**
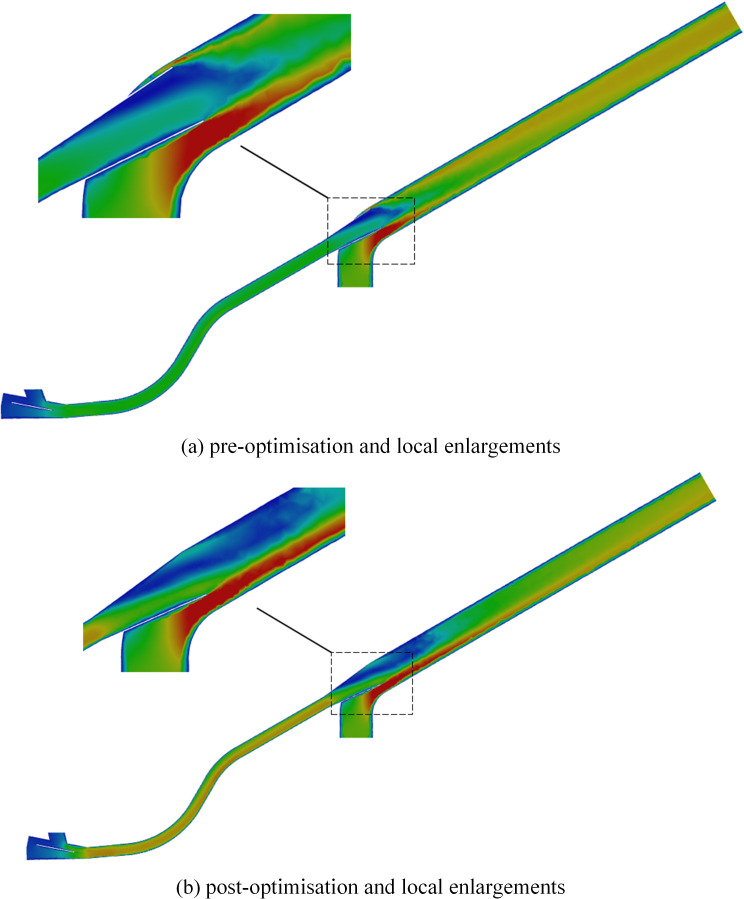
Comparison of velocity clouds before and after structural optimisation. (a) pre-optimisation and local enlargements. (b) post-optimisation and local enlargements.

The model was reconstructed using the optimized structural parameters, and simulation analyses were conducted accordingly, the velocity distribution results after optimizing the throat inlet diameter are shown in Fig 20.

As shown in the figure, the acceleration zone created by the reduced throat inlet diameter compresses the boundary layer’s momentum thickness, suppresses the formation of large-scale separation vortices, and significantly reduces both the velocity gradient variation and vortex intensity at the throat outlet. The reduced phase difference in the velocity field leads to a shift of flow instability eigenfrequencies toward higher frequency ranges. The improved continuity of flow curvature confirms the enhanced resistance of the boundary layer to separation. The helical flow field generated by the increased seed incidence angle induces a self-sustained vortex ring structure in the vertical direction, effectively mitigating energy dissipation caused by the convergence of vortex cores. Additionally, the cyclonic component introduced by the helical flow reduces the relative contribution of viscous dissipation, enabling rolling friction between particles to replace collisional fragmentation as the dominant energy dissipation mechanism.

**Fig 21:**
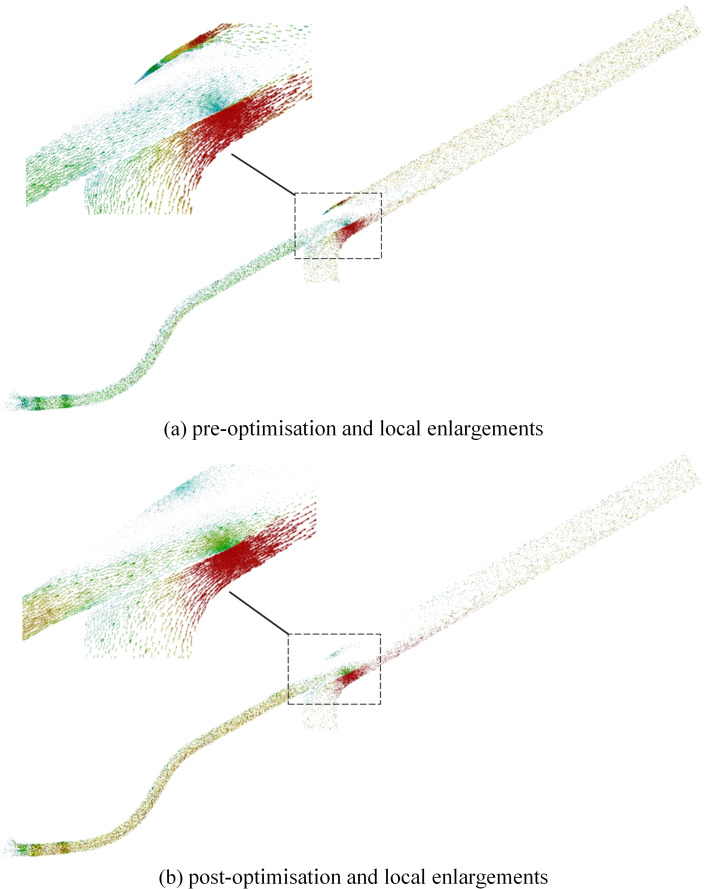
Structurally optimised velocity vector. (a) pre-optimisation and local enlargements. (b) post-optimisation and local enlargements.

As show in Fig 21, in the original structure, significant velocity stratification is observed at the throat outlet, whereas the optimized configuration exhibits a smooth color gradient and a marked increase in velocity amplitude, confirming dynamic compatibility between the expansion section curvature and the velocity field. The improved alignment of velocity vectors in the optimized case indicates compression of the boundary layer’s momentum thickness, effectively suppressing the formation of horseshoe vortices. High-vorticity regions present in the original flow are eliminated post-optimization, indicating a transition of vortex transport from random pulsation to organized dissipation, with energy dissipation concentrated in the viscous-dominant regime via vortex stretching and compression. Furthermore, the introduction of a helical streamline structure due to increased seed incidence angle establishes a self-stabilizing flow field through centrifugal force–pressure gradient coupling, significantly reducing transverse pulsations compared to the axisymmetric flow in the original design.

## Conclusion

Based on bench tests and research data analysis, this study emphasizes the significance of optimizing the design of pneumatic seeders. It proposes several improvement strategies, including optimizing the throat outlet and inlet diameters, analyzing the flow field variations within the pneumatic conveying system, adjusting the seed entry angle, and evaluating its impact on the collision frequency between seeds and the tube wall. The study then identifies optimal structural parameters to enhance system performance. The main conclusions are as follows:

This study optimized the throat outlet diameter to 58 mm, analyzing its effect on velocity within the pneumatic convey system. The optimization achieved maximum velocity at the throat outlet’s center, effectively reducing eddy currents and minimizing the probability of blockages in the seed delivery system.

This study investigates the impact of varying throat inlet diameters on vortex flow. The results indicate that adjusting the throat inlet diameter had little effect on vortex flow compared to the original machine design. This study investigates the impact of varying throat inlet diameters on vortex flow. The results indicate that adjusting the throat inlet diameter had little effect on vortex flow compared to the original machine design. Consequently, altering the throat inlet diameter does not effectively address the vortex issue at the throat outlet.

The study examined how optimizing the seed entrance inclination angle affects seed-wall collisions in a pneumatic convey system. After adjusting the angle, a reduction in particle-wall collisions was observed at α = 77°, indicating improved interaction between seeds and the seeding mechanism. This improvement could help reduce seed breakage to some extent.

This study utilizes numerical simulation and experimental methods to optimize the structure of the pneumatic conveyance system in pneumatic seeders. The goal is to address existing issues related to internal seed blockage and breakage, thereby establishing a foundation for the design and application of pneumatic seeders.
